# Incidental Prostate Cancer Detected in Holmium Laser Enucleation of the Prostate (HoLEP) Specimens: A Single-Center Experience

**DOI:** 10.7759/cureus.90916

**Published:** 2025-08-24

**Authors:** Momen Sid Ahmed, Nkwam Nkwam

**Affiliations:** 1 Department of Urology, King's College Hospital NHS Foundation Trust, London, GBR

**Keywords:** active surveillance, benign prostatic hyperplasia, gleason score, histopathology, holep, holmium laser enucleation of the prostate, incidental prostate cancer, prostate volume, psa density, turp

## Abstract

Background: Incidental prostate cancer (iPCa) is an unsuspected malignancy found in tissue resected for benign prostatic hyperplasia (BPH). We evaluated the prevalence and characteristics of iPCa among patients undergoing holmium laser enucleation of the prostate (HoLEP) at our center.

Methods: We retrospectively reviewed 259 consecutive HoLEP cases. Patients with known prostate cancer before surgery were excluded from iPCa rate calculations. Demographics, prostate-specific antigen (PSA), prostate volume, and histopathology were recorded. Between-group comparisons were performed using the Mann-Whitney U test and the chi-square test (p < 0.05).

Results: iPCa was identified in 37/259 (14.3%) cases. Gleason distribution among iPCa was 3+3=6 in 23/37 (62.2%), Gleason 7 in 9/37 (24.3%) (3+4=7 in eight; 4+3=7 in one), Gleason 8-10 in 4/37 (10.8%), and not assigned in 1/37 (2.7%). Patients with iPCa were slightly older (median age, 75 years) than those with benign histology (median age, 72 years; p=0.014). Median PSA (7.85 vs. 7.20 ng/mL; p=0.45), prostate volume (112.5 vs. 120 cc; p=0.25), PSA density (0.069 vs. 0.061 ng/mL/cc; p=0.29), and proportions with PSA>10 ng/mL (11/37 (29.7%) vs. 65/222 (29.3%); p=0.998) or prostate volume >100 cc (23/37 (62.2%) vs. 147/222 (66.2%); p=0.891) did not differ significantly.

Conclusion: Approximately one in seven HoLEP patients (37/259, 14.3%) had iPCa, predominantly of the low-grade (23/37, 62.2%) type. Most cases were suitable for conservative management; only 4/259 (1.5%) of the entire cohort had incidentally detected high-grade (Gleason ≥8) disease.

## Introduction

Incidental prostate cancer (iPCa) has long been observed in tissue resected for benign prostatic hyperplasia (BPH), with prostate-specific antigen (PSA)-era TURP (transurethral resection of the prostate) series reporting lower detection rates than earlier eras [1,2]. Following the widespread adoption of PSA testing, iPCa detection at TURP decreased substantially [3]. Holmium laser enucleation of the prostate (HoLEP) removes the adenomatous component of the prostate en bloc and is effective across prostate sizes [4], and guideline bodies recognize size-independent use and contemporary management pathways for BPH and prostate cancer [5,6].

A recent systematic review of endoscopic enucleation procedures summarized the incidence range, predictive factors, and outcomes of iPCa after enucleation [7]. Contemporary HoLEP cohorts have further detailed rates and risk factors (including operative and pathological predictors) [8-11], and recent data have also described natural history after HoLEP [12] and the prevalence of iPCa in national datasets and temporal trend analyses [13,14]. Outcomes following TURP-detected iPCa, based on the initial management strategy, have also been described [15]. Additionally, comorbidity-linked predictors for iPCa after HoLEP have been explored (e.g., diabetes) [16]. The background reservoir of latent, microscopic prostate cancer in older men provides important biological context for iPCa [17], while classic PSA-era studies outline progression risk in stage T1a disease and management outcomes for incidental carcinoma treated definitively [18,19]. Pathology sampling considerations when minimal cancer is identified on TURP are also relevant when characterizing incidental disease [20]. We report our single-center HoLEP experience, quantifying iPCa prevalence/grade and comparing simple clinicopathological variables.

## Materials and methods

Study design

This is a single-center, retrospective observational study of consecutive HoLEP procedures for BPH conducted over a five-year period.

Study population and sample size

All adult men undergoing HoLEP for symptomatic BPH were included (N=259). Men with a known diagnosis of prostate cancer before HoLEP were excluded from iPCa prevalence estimates and from between-group comparisons; the prevalence denominator therefore comprised all eligible HoLEP cases without known cancer (n=259 in this cohort). All available cases were included (no a priori sample size calculation).

Study measures

The preoperative variables included age, ASA (American Society of Anesthesiologists) grade, PSA, and ultrasound-estimated prostate volume. HoLEP was performed using low-power Ho:YAG with anatomical enucleation of the adenomatous tissue as appropriate and intravesical morcellation [4]. All tissue was submitted for histopathology. iPCa was defined as adenocarcinoma detected on HoLEP specimens in men not previously diagnosed (pathologic T1a/T1b). Where reported, Gleason score and tumor volume comments were extracted.

Ethics statement

Institutional approval was obtained with a waiver of consent, as the analysis involved a retrospective review of de-identified data.

Statistical analysis

Continuous variables are summarized as median (interquartile range, IQR) and compared using the Mann-Whitney U test. Categorical variables are analyzed with the chi-square test (or Fisher's exact test, where appropriate). Two-sided p < 0.05 denotes significance.

## Results

Cohort characteristics

The mean age of the patients was 72.4 ± 7.8 years; the median PSA level was 7.38 ng/mL (IQR, 4.23-12.52); the median prostate volume was 119 cc (IQR, 92-152 cc). The following were the ASA grades: 26/259 (10.0%) ASA-1, 160/259 (61.8%) ASA-2, 72/259 (27.8%) ASA-3, 1/259 (0.4%) ASA-4. Indications included LUTS (lower urinary tract symptoms) 131/259 (50.6%), acute urinary retention 64/259 (24.7%), high-pressure chronic retention 62/259 (23.9%), and low-pressure chronic retention 4/259 (1.5%) (patients could have more than one).

Incidental cancer rate and pathology

iPCa was identified in 37/259 (14.3%). Gleason distribution: 3+3=6 in 23/37 (62.2%), Gleason 7 in 9/37 (24.3%) (3+4=7 in 8; 4+3=7 in 1), Gleason 8-10 in 4/37 (10.8%), not assigned in 1/37 (2.7%).

Comparison of iPCa vs. benign histology

Baseline characteristics based on pathology are summarized in Table 1. Patients with iPCa were slightly older (median age, 75 vs. 72 years; p=0.014). The median PSA (7.85 vs. 7.20 ng/mL; p=0.45), prostate volume (112.5 vs. 120 cc; p=0.25), and PSA density (0.069 vs. 0.061 ng/mL/cc; p=0.29) were not significantly different. Proportions with PSA>10 ng/mL (11/37 (29.7%)) vs. (65/222 (29.3%); p=0.998) and prostate volume >100 cc (23/37 (62.2%)) vs. (147/222 (66.2%); p=0.891) were similar.

**Table 1:** Baseline characteristics by pathology (HoLEP cohort, N=259). Values are presented as median (IQR) or n (%). PSA: prostate-specific antigen; iPCa: incidental prostate cancer

Variable	Benign Histology (n=222)	iPCa (n=37)	p-value
Age, years	72 (66–77)	75 (70–79)	0.014
PSA, ng/mL	7.20 (4.10–12.40)	7.85 (5.58–12.80)	0.45
Prostate volume, cc	120 (93–155)	112.5 (85.5–131.8)	0.25
PSA density, ng/mL/cc	0.061 (0.038–0.101)	0.069 (0.048–0.117)	0.29
PSA >10 ng/mL	65/222 (29.3%)	11/37 (29.7%)	0.998
Prostate volume >100 cc	147/222 (66.2%)	23/37 (62.2%)	0.891

Follow-up and initial management

Most iPCa were low risk; active surveillance/observation was recommended in the great majority (reflecting guideline-concordant practice) [5,6,9,12]. Patients with high-grade findings (Gleason ≥8; 4/37 (10.8%)) underwent further staging and individualized counseling; definitive therapy was chosen in selected cases.

## Discussion

Our iPCa rate after HoLEP (14.3%) lies toward the upper range for endoscopic enucleation (pooled: ≈8%, reported range: 2.5-23%) [7]. By contrast, contemporary PSA-era TURP series often report a rate of approximately 2-5% [3]. Variability likely reflects tissue yield, case mix, and local preoperative pathways. Multiple HoLEP cohorts detail independent predictors (e.g., age, PSA density, resected weight) with mixed reproducibility [8-11], and natural history and long-term outcome data support conservative management for most low-risk iPCa after HoLEP [9,12]. National-scale analyses and temporal trends reinforce that iPCa remains a non-trivial finding in BPH surgery [13,14]. TURP-based series provide context for outcomes based on initial strategy [15], and comorbidity-linked risk (e.g., diabetes) has been proposed as a predictor of iPCa after HoLEP [16]. Autopsy studies highlight the high background prevalence of latent, microscopic disease in aging men [17]. In the PSA era, progression predictors in T1a disease have been outlined [18], and selected incidental cancers treated with radical prostatectomy demonstrate generally favorable outcomes [19]. Pathology sampling nuances remain pertinent when minimal cancer is identified on TURP, particularly in staging/quantification discussions [20]. Additionally, a contemporary narrative review specifically focused on iPCa after HoLEP synthesizes detection rates and practical follow-up considerations, complementing these findings [21]. Further technique comparisons (e.g., aquablation vs. HoLEP) show differing perioperative iPCa rates in some series [22], while broader systematic syntheses across BPH surgeries contextualize prevalence and risk factors [23].

Placing our findings into a sharper context: in a contemporary HoLEP cohort, Porto et al. identified clinical and pathological factors associated with iPCa and reported surgical outcomes that mirror our experience, namely, a predominance of low-grade, organ-confined tumors and preserved perioperative safety [8]. Earlier single-center HoLEP series consistently found that most incidental cancers are GG1-2, with low short-term oncologic event rates under observation [9-11]. Our grade distribution (Gleason 3+3=6: 62.2%; Gleason 7: 24.3%, predominantly 3+4=7; high-grade ≥8: 10.8%) is typical and closely aligns with these reports [9-11]. Natural-history data specific to HoLEP further support conservative initial management for the majority of iPCa: Han et al. observed that most patients remained on surveillance without progression at mid-term follow-up [12], while Tominaga et al. described favorable long-term urinary and oncologic outcomes after HoLEP-detected iPCa [9]. These observations are consistent with guideline frameworks that favor active surveillance for low-risk localized prostate cancer and recommend risk-adapted imaging/biopsy to guide escalation [5,6].

With respect to detection rates across BPH procedures, technique and tissue retrieval matter. Endoscopic enucleation (including HoLEP) tends to yield larger tissue volumes than chip-based resections, which plausibly increases the opportunity to uncover small foci of carcinoma [7]. Conversely, aquablation generally retrieves less tissue; comparative data show lower perioperative iPCa detection versus HoLEP, highlighting a potential diagnostic trade-off between minimally invasive resection and histologic yield [22]. Meta-analytic syntheses that pool different BPH surgeries confirm wide heterogeneity driven by preoperative workup, pathology processing, and center practices [7,23]. Our center does not mandate routine pre-HoLEP biopsy/MRI for every elevated PSA, which likely contributes to a higher iPCa rate than series with stringent preoperative exclusion of cancer [13,14]. Importantly, this does not imply worse oncologic outcomes: TURP-era data show that progression risk for T1a disease is generally low under modern surveillance paradigms [18], and when definitive therapy is indicated, outcomes can be excellent in appropriately selected patients [19].

Predictor signals remain inconsistent across studies. Some cohorts have implicated PSA density and smaller gland size as risk markers [10,11], while others report comorbidity-linked associations such as diabetes [16]. In our analysis, age differed modestly (with older individuals in iPCa), whereas baseline PSA, prostate volume, and PSA density did not; such variability is common in the literature and likely reflects differences in biopsy/MRI use, referral patterns, and sample size [7-11,16]. From a pathology standpoint, comprehensive submission and sampling of all enucleated tissue is essential to stage small-volume disease reliably and to minimize under-detection [20,21]. Finally, population-level and time-trend data suggest that iPCa rates may decline as preoperative MRI and targeted biopsy become more widespread [13,14], but HoLEP's full-adenoma enucleation continues to provide a robust opportunity for incidental cancer detection when it remains present.

Strengths include complete single-center capture, large gland sizes typical for enucleation, and whole-specimen submission. Limitations include the retrospective design, one ungraded malignant case, and limited oncologic follow-up; residual confounding (e.g., by screening intensity) cannot be excluded. Future work should integrate systematic preoperative MRI/biopsy pathways and standardized PSA density reporting to refine prediction models and to determine whether any functional differences persist after adjustment.

## Conclusions

In a consecutive HoLEP cohort of 259 men, iPCa occurred in 37 (14.3%), predominantly of low grade (23/37, 62.2%). Most cases were suitable for conservative management; only 4/259 (1.5%) had incidentally detected high-grade disease. Routine histopathological assessment and preoperative counseling about iPCa remain essential parts of HoLEP care pathways.
